# Emptiness in the study of emotions in the teaching-learning process of reading-writing during the COVID-19 pandemic

**DOI:** 10.3389/fpsyg.2022.991574

**Published:** 2022-12-21

**Authors:** Mariacarla Martí-González, Angel Barrasa, Simone Belli, Johana Espinel, Silvia Da Costa, Caridad López-Granero

**Affiliations:** ^1^Department of Education, University of Cantabria, Santander, Spain; ^2^Department of Psychology, University of Zaragoza, Zaragoza, Spain; ^3^Department of Social Anthropology and Social Psychology, Complutense University of Madrid, Madrid, Spain; ^4^Department of Psychology, State University of Milagro, Milagro, Ecuador

**Keywords:** emotions, reading-writing learning, COVID-19, virtual contexts, education

## Abstract

The teaching-learning process of reading and writing has great relevance in the psycho-emotional and socio-psychological development of school-age children. This is an exercise in which they develop imagination, attention and memory capacity and through this process the management of emotions and sensitivity and capacity of apprehension of reality. The crisis derived from the COVID-19 pandemic transformed reality in an unprecedented way in the recent history of humanity and the educational context was shaken by all these changes. With confinement, the teaching-learning process of reading and writing, which is designed to take place in person, had to be developed in a hybrid or online way, which was a major challenge for teachers and families and, of course, also for children who were in the process of learning. One of the aspects that was blurred in this context was the role of the teacher, which in this case is one of the most important elements, to achieve adequate learning of reading and writing. On one hand, the teacher is in charge of helping to manage the emotions derived from the learning process itself and, on the other hand, he is a key figure so that this is not only technical learning but also contributes to the child’s socio-emotional development. The aim of this study was to analyze the impact of the socio-psychological changes that have occurred in the educational context since the COVID-19 pandemic on the emotions linked to the teaching process, learning of reading and writing through a systematic review of the studies carried out on the subject, in order to provide recommendations for face-to-face learning in the post-COVID-19 era. A systematic review strategy was devised and the literature search was conducted. The search was conducted using ERIC, Dialnet, Scopus, WoS, EBSCO, and Google Scholar databases. This systematic review took place during the month of May 2022. The results show that given the scarce presence of empirical studies on the subject, the objective has only been partially met. However, a systematic review of the studies carried out on the subject. For the identification of recommendations in the development of face-to-face learning in the post-covid era, it has been possible to identify some ideas of interest for future curricular designs in primary school students who are immersed in learning to read and write.

## Introduction

### Teaching-learning process of reading and writing

The teaching-learning process of reading-writing is anything but simple and plain since it involves not only the recognition of written signs but also the identification of the connotations of said signs in various fields, scenarios and situations -on one hand- and the codification in these signs of one’s own thoughts, feelings, emotions, desires, etc. Molina (1991) defines reading-writing as a process from which the identification and consequent writing of texts occurs, to acquire posteriori the ability to understand and elaborate them.

Grinstead et al. (2014), consider that the global reading process can be subdivided into several sub-processes such as the ability to decipher written words, to be able to express them orally and to be able to understand the way of expressing oneself and the intention of the author of the written message.

In any case, reading and writing involves learning to decode a set of signs based on their denotations and connotations and the encoding of these signs for the expression of certain ideas. According to Auzias (1978), literacy is a way of expressing language through communication based on changing symbols and signs, since these signs and symbols evolve to the extent that civilizations do.

For the acquisition of reading and writing in Primary Education classrooms, various methodological procedures are used that are based on the concepts of ascending and descending information processing. The selected methodological variant will depend on the evaluation of the previous knowledge of the students and the sensitivity for the reading-writing work. Some of the most used methods would be:

(a) Methods focused on teaching-learning, ascending process: analytical methods.

(b) Methods focused on learning-teaching, which are oriented from the significance of the descending process: synthetic or global methods.

(c) Mixed process methods: they combine the proposals of the analytical and synthetic methods, with all the possible variants. They arise to establish a bridge between the interpretations of the two learning models and seek to synthesize and bring together the two assumptions: ascending and descending.

The methods that are organized according to the conception of teaching as bottom-up processing are the analytical methods. Alphabetical, phonetic, syllabic, and onomatopoeic are distinguished. In all of them, the object of learning (reading-writing) is analyzed, starting from graphic units and their phonetic emission (letters, phonemes, syllables or sounds associated with gestures), and unions are established between them to compose the syllables (at first simple, then inverse and linked), and to form the possible words that result from the serialized spellings (LaBergue and Samuels, 1974).

The ascending model is the one in which the person begins the reading process by the letters and their groups, in a process that increases until the reader manages to understand the larger units, the words and the complete text (LaBergue and Samuels, 1974). The model focuses on the text and is only based on decoding. This model is based on traditional theory, and posits that understanding is achieved through sequential and hierarchical learning of a series of visual discriminations.

Cuetos Vega (2000) explains, through the ascending model, that reading is made up of perceptual, lexical, syntactic and semantic processes in that order, so the process begins when the reader uses his senses to “extract” information graphic signs.

On the other hand, there is the top-down model, which is based on the notion that, in addition to the existence of the text and its decoding, the experiences, knowledge and prior learning of children must be considered (Goodman, 1971; Smith and Goodman, 1971). In this way, from his/her previous experience, the reader makes hypotheses and inferences previously to reading, which make possible the textual processing in order to contrast its verification. According to this model, learning to read would imply not so much the sequential acquisition of a series of discriminative responses, but rather the learning and use of prior syntactic and semantic knowledge to anticipate the text and its meaning (Smith and Goodman, 1971).

A fundamental role in the reading-writing process is played by phonological awareness, which has been conceptualized as the metalinguistic ability that allows us to reflect on oral language, specifically, it would be the ability to make judgments about the sounds of the language itself (Jennifer and Cannock, 2014). Phonological awareness develops, fundamentally between 3 and 7 years of age (Etchepareborda and Habib, 2001), and is divided into levels based on difficulty (Defior and Serrano, 2011):

(a) Lexical awareness: Ability to identify the words that make up sentences and deliberately manipulate them. (How many words are there in a sentence?)

(b) Syllabic awareness: Ability to segment and manipulate the syllables that make up words. (How many syllables are there in a word?).

(c) Intra-syllabic awareness: Ability to segment and manipulate the start (consonant/s before the vowel) and the rhyme (the vowel and consonants that follow) of the syllables. An example would be asking about the difference between “gol” and “col” (different start) or between “sol” and “salt” (different rhyme).

(d) Phonemic awareness: Ability to segment and manipulate the smallest units of speech that are phonemes. (How many sounds are heard in a word?)

A very important role in the acquisition of phonological awareness and, therefore, in the teaching-learning process of reading and writing is played by the link with significant others for the students (family, peers and teachers). If this occurs in face-to-face contexts, its importance increases in teaching in virtual contexts in crisis situations. In fact, Weber et al. (2021) in a study whose objective was to examine whether social and ethnic disparities in the reading achievement of primary school students widened during COVID-related school closures in spring 2020 and whether increased disparities are mediated by parental involvement in distance learning; explain the mediating role of parental involvement in distance learning in these specific circumstances. Thus, the authors found evidence that parents with high socioeconomic status show more favorable (structuring participation) and less harmful (intrusive participation) forms of participation in distance learning (Weber et al., 2021). This is consistent with the results of other investigations, such as the case of Sander et al. (2021) who developed a study on parental involvement during distance learning related to COVID-19.

The bonding processes develop the emergence of basic emotions that could make possible and facilitate in some cases or hinder in others the teaching-learning process of reading and writing through the development of phonological awareness.

### The role of emotions in the teaching-learning process of reading and writing

Emotions play an absolutely relevant role in everyday life since in many cases they mediate, moderate and in any case participate in the relationship established with reality, be it with links, objects, situations, contexts, etc. According to Bisquerra (2003) there is more or less agreement in considering emotion as a complex state of the organism, which predisposes to an organized response from an excitation or disturbance. Thus, emotions are generated in response to an external or internal event (Bisquerra, 2003). In general, we can speak of three components in an emotion: neurophysiological, behavioral, and cognitive. The neurophysiological component is expressed in responses such as tachycardia, sweating, vasoconstriction, hypertension, muscle tone, flushing, dry mouth, changes in neurotransmitters, hormonal secretions, breathing, among others. These are all involuntary responses that are beyond the subject’s control. From the behavioral component, the type of emotion that is being experienced by the subject can be inferred (facial expressions, non-verbal language, tone of voice, volume, rhythm, body movements). Thus, the cognitive component or subjective experience is what is sometimes called feeling (Bisquerra, 2003).

This author relates the three components with the classification of didactic objectives: a) “Facts, concepts and conceptual systems” with the cognitive dimension; b) “Procedures” with behavior; c) “Attitudes, values and norms” with respect to the emotional dimension (Bisquerra, 2003).

In the case of the reading and writing processes, it does not happen differently. In fact, it could be said that these processes are important generators of emotional experiences. Pekrun (2022) analyzes eight studies that address the issue of emotions in reading and learning from texts and concludes that three fundamental messages emerge from this set of studies.

First of all, reading can generate strong emotions, which are essential in understanding the processes and effects of reading on learning. In this sense, the author himself specifies that emotions can promote reading and comprehension, however they also have the power to reverse the positive effects of reading texts, which leads to a reduction in conceptual progress and a change in attitude (Pekrun, 2022).

Second, it is concluded that different emotions can exert different effects. In principle, in global learning the effect of positive emotions (i.e., pleasant) is positive and the effect of unpleasant emotions is negative (Barroso et al., 2020; Loderer et al., 2020; Camacho-Morles et al., 2021). However, in light of these specific studies, to conclude that positive emotions are always beneficial and negative emotions always detrimental could be questionable. Pekrun (2022) cites the examples of studies by Mensink (2021) in which happiness was a negative predictor of performance, and Dever et al.’s (2021) research, in which the results indicated that combinations of positive and Negative emotions proved to reduce performance.

As a third and last element, it is pointed out that it is also important to acknowledge that emotions influence different stages and levels of processing information from texts.

It occurs in a similar way in the case of the teaching-learning processes of reading and writing, where this dimension is integrated with the cognitive processes and strategies associated with said learning.

### Differences in teaching-learning processes in the digital context vs. face-to-face context

The online teaching-learning process involves the deconstruction of the traditional educational situation, which makes the process more complex, since it is not just about adapting content and tasks to the new modality, but there are other psychosocial and educational processes such as the link between students and teachers that must be resized and relocated in the new situation. Following the previous example, in face-to-face the space for this link to fulfill its psychological and educational function is guaranteed (even if it may or may not develop adequately). However, in blended, virtual, or online modalities this space must be created and open the possibility of a close and potentially constructive interrelation. This relationship is the very basis of the educational situation.

It is then a question of working on language, considering that the colloquially of speech must be able to be expressed in virtual communication channels, just to cite an example of the most significant elements. In the same way, practical activities, content application, reflection, etc.; they must maintain methodological rigor to guarantee student learning, but it is also important that they be designed considered the real physical distance that marks this teaching modality.

Another very important element is the care of the teacher-student bond, which is the basis of the educational situation. Taking into account these two previous elements and linking them with Vygotskian concepts such as the Zone of Proximal Development (ZPD) and the experience, we can establish the communication process in the academic context, specifically in the classroom space, is one of the main elements of the nuances of this exchange that can be used, both in non-verbal aspects and in the organization and intentionality that is reflected in the discourse.

This link is, in the online space, one of the elements that must be attended to with more care. Teachers are communicators and as such, care must be taken that, from the position of issuer, the message arrives clearly and precisely, and that our concern and empathy is reflected with the situation in which the student is also faced with the anxieties that these changes entail.

These, for example, would be concepts that should be analyzed (ZPD and experience) and that are key to guaranteeing not only the transmission or even the construction of a certain knowledge, but also the true value of education. Perhaps here lies one of our main challenges in this situation, in this new scenario, and it is the fact that the transformations of the teaching-learning process cannot take our eyes away from the fact that the main thing is education. The core element is that this teaching-learning process of a discipline must be accompanied and sustained by the ethical and humanistic values that the teaching work must transmit to the new generations and that is our commitment in the day to day of our profession. The teaching-learning process must place education as a leading element.

In this sense, Borroto and Rebollar (2015) point out that among the great challenges of the contemporary world is the education and development of competent professionals, capable of facing scientifically and with humanistic ethics, problems and conflicts related to the healthy satisfaction of educational needs of today, through the cooperative act, in collaborative relationships and with autonomy.

Previous issues suppose a challenge for education. In the case of distance teaching-learning processes it becomes an unavoidable challenge that it must place at the center of research analysis and action.

### COVID-19 pandemic contextualization

In March 2020, the reality of daily life changed drastically in all spheres, areas and levels throughout the world. Very few regions, countries and/or spaces of reality remained unmoved by the shock caused by the arrival of COVID-19.

Education has always been a vulnerable scenario in the face of social, political and economic changes in history. Social dynamics impact educational processes at all levels, often to the detriment of the quality of teaching, the appreciation of the teacher’s professional role, and the necessary and vulnerable link with students.

The crisis derived from the COVID-19 pandemic has come to transform daily life: these have been and will be very difficult times in which this new reality imposes challenges of various sizes that put physical, mental and social capacities to the test. Uncertainty has been imposed as a variable to manage even in the most basic and simple issues. Lives have been lost, the organization of multiple processes in all spheres of society has had to be reconsidered in record time and new demands have had to be assumed without, on occasions, having the minimum conditions created for it. At the time of global confinement, working families from various sectors were faced with the immense and extremely complex task of making family reconciliation possible in the face of the arrival of teleworking or, in the worst scenario, assuming unemployment. Health professionals have pushed their bodies and psyches to the limit, workers have fought from invisibility to guarantee basic services without – on many occasions – being recognized for it; and all of the above, just to cite some of the most significant examples.

In the field that occupies the present work -education-, faculty at all levels of education have been seen reorganizing and adapting methodological plans for half an academic year to adjust to online teaching, while managing changes at the family level. Students -also of all levels-, have had to deal with the resistance and anxieties derived from a radical change in teaching, without being fully aware of the process they were experiencing. The reality is no longer what was known before the pandemic and in the face of this, various challenges have been considered and posed. One of the main ones has had to do with the teaching-learning process of reading and writing in the context of confinement and subsequent development of classes in a virtual, hybrid context and even in face-to-face space but with restrictions derived from the sanitary situation.

The truth is that the questions that have been developed in previous paragraphs have been addressed since the start of the pandemic and attempts have been made to develop alternatives to guarantee compliance in a more or less purposeful and conscious manner. In other words, when it was necessary toward the beginning of 2020 to change from a face-to-face teaching modality to the different variants of distance learning, the first questions revolved around how to adapt materials and activities to the new ways of teaching and how to maintain the educational bond –which is to say human- with the student. However, the speed and immediacy demand of this process neglected the professional role of the teacher and the teacher-student bond. This, for example, is an essential element, the teacher-student bond is one of the key resources of the teaching-learning process and has an impact on the success of this process. Indeed, it would be interesting to explore the impact of the pandemic on this very fundamental link. Ramkissoon (2022) develops and underlines the importance of social bonds in the construction of a more positive attitude to life, about the COVID-19 pandemic through commonly shared meanings. The paper itself analyzes how people’s resilience capacity, as well as emotional well-being, social and emotional psychological empowerment in challenging times, will be influenced by the link that exists between people, whether digitally or in person (Ramkissoon, 2022). So, this must be an unavoidable object of analysis.

The result was a vertiginous inertia in which more time was often devoted to technical, technological, and operational issues than to the very approach of the healthy limits for the performance of the role of teacher or the bond with the students. These two elements are essential for the teaching process of reading and writing, since it is precisely these two elements that allow us to contain and manage the emotions that emerge in this teaching-learning process and, in addition, to enhance the classroom emotions that are inherent to true meaningful learning.

This framework is an opportunity for unprecedented research if we consider that much of what is currently known regarding the role of emotions in the literacy learning process has been investigated under normal conditions. Even in many cases certain experiments have been limited for ethical reasons since it was not possible to significantly alter certain academic and school dynamics. So that most of the production of knowledge in the area -at least from psych pedagogy- has been developed from normal conditions, which presents us with a scenario of exceptional possibilities to analyze what happens when the given conditions do not are ideal or when there are deficiencies of various kinds.

Specifically, it is an ideal time to compare the role of emotions in the teaching-learning processes of various subjects and obtain data of incalculable axiological, epistemological, theoretical and - fundamentally - methodological value, despite even the haste and difficulties in the which online teaching has emerged in this case. So, a first step would be to know the emotions that have been involved in the teaching-learning process of reading and writing during the last 2 years.

This way, the following initial research questions are presented:

Research Question 1: Have the changes that have occurred in educational institutions, derived from the COVID-19 pandemic, influenced the emotions linked to the teaching-learning process of reading and writing?

Research question 2: What impact has the COVID-19 pandemic had on emotions linked to the teaching-learning process of reading and writing?

An outline of the above is what this article intends. To answer the above questions, the objective of this study has been defined: to analyze the impact of the socio-psychological changes that have occurred in the educational context since the COVID-19 pandemic on the emotions linked to the teaching-learning processes of reading and writing, through a systematic review of the studies carried out on the subject; to provide recommendations for face-to-face learning in the post-COVID-19 era.

## Materials and methods

### Study design

A systematic review strategy was devised and the literature search was conducted. The search was conducted using University of Zaragoza, University of Cantabria and European University from Atlantic’s subscription to access the following databases: Education Resources Information Center (ERIC), Dialnet, Scopus, Web of Science (WoS), EBSCO, and Google Scholar. This systematic review took place during the month of May 2022 and the methodological design is a review study, following the PRISMA guideline.

The subject and keyword searches were conducted in five parts.

Emotions and their cognate terms:

Emotion OR Feeling OR Emotions OR Feelings OR Emotional Reaction OR Emotional AND.

Reading-Writing Learning and its cognate terms:

Writing-Learning OR Learning OR Primary School Learning OR Elementary School OR Reading Learning OR Writing Learning AND.

Learning in Virtual Context and its cognate terms:

Learning in Virtual Context OR Learning in Digital Context OR Virtual Context Learning OR Digital Context Learning AND.

Factors and variables:

Emotions OR Affective Learning OR Reading-Writing Learning OR …

The Boolean operators (OR/AND) and search filters were applied to obtain more focused results. The articles included in the final search were peer-reviewed and the references of publications sourced from these searches were hand searched to obtain additional abstracts.

Regarding the Inclusion and Exclusion Criteria, only peer-reviewed articles published within the last 2 years (2020–2022) and with full text available were included.

**Figure 1:**
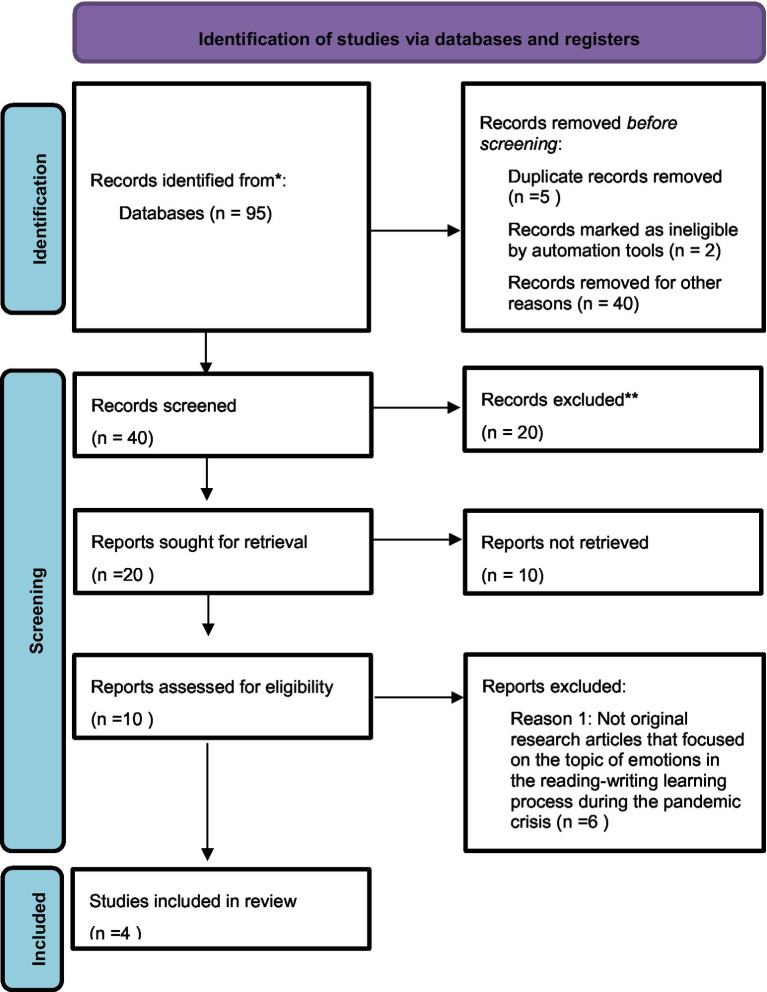
Decantation chart of the studies.

Studies included in the final analysis were original research articles that focused on the topic of emotions in the reading-writing learning process during the pandemic crisis. It was also considered as a topic to analyze the teaching-learning process of primary school students, since it is precisely in the first cycle of this period where the learning process of reading and writing takes place and, necessarily, an analysis of this stage implies analyzing said process (Figure 1).

## Results

The studies included in the final selection were analyzed following a qualitative analysis paradigm. From the initial population of 95 articles, a sample of 4 articles was defined following the inclusion/exclusion criteria explained in the previous section (Table 1).

**Table 1:** Description of the articles included.

RQ	Author/Tittle	Year	Aim	Participants	Method	Results
1 and 2	José Jesús Sánchez Amate, Antonio Luque de la Rosa, Rafaela Gutiérrez Cáceres and Alejandro Vargas Serrano/The Effects of COVID-19 in the Learning Process of Primary School Students: A Systematic Review	2021	This review addresses the repercussions of COVID-19 at the educational level in the primary education stage, delving into the effects produced in teaching and different aspects related to it, such as the situation and challenges of teachers, family involvement, and the perceptions and repercussions of the learning and socio-educational development of students (especially in the case of students with Autism Spectrum Disorder).	103 articles	Methods: The methodological design is a systematic review study, following the PRISMA guidelines, from a search carried out during the month of July 2021 in the Scopus, Dialnet, and WoS databases on the object of study.	The research reveals the presence of an existing digital gap in certain sectors of the student population aggravated by the pandemic, as well as the scarcity of general teacher training in this type of situation, leading to different personal and professional problems that hinder teaching and emphasize the vulnerability of the right to education, which leads to further promoting the already existing social inequalities.
1 and 2	Lisette Cavallo/ THE CHALLENGE OF DEVELOPING THE TEACHING PROCESS AND LITERACY LEARNING IN PANDEMIC.	2021	To identify the factors that intervene in the process of teaching and learning of literacy in the context of a pandemic by COVID-19, through the analysis of research and perspectives theoretical on this subject, requiring the bibliographic review of study material from journals indexed in Education and Science Social published on the web.	A review of the literature on the subject was carried out.		It is concluded that it is necessary understand, from a theoretical-interpretative perspective, the factors that are currently involved in achieving the competencies of literacy recognizing its implication in the educational process with the purpose of developing an emergent literacy from the first years of life of boys and girls to enhance these skills.
1 and 2	Almut E. Thomas/ First and Second Graders’ Reading Motivation and Reading Comprehension Were Not Adversely Affected by Distance Learning During COVID-19	2021	To investigate differences in reading comprehension and self-determined reading motivation of students who attended grades one and two during or before the pandemic.	206 elementary students	This study used a quasi-experiment with 206 elementary students to investigate differences in reading comprehension and self-determined reading motivation of students who attended grades one and two during or before the pandemic.	The results revealed no differences in reading comprehension and reading motivation between the groups, contradicting the assumption that the pandemic-driven shift to distance learning would inevitably impair young students’ achievements and self-determined motivation.

The first element that is interesting to draw attention to is the fact that there are few studies -at least published in journals indexed in the databases in which the search was carried out- on the role of emotions in the teaching process—learning of reading and writing in the last 2 years in which they try to answer the questions of this article. In fact, only one article was found during the research with the specific topic that was intended to be addressed and this was not empirical in nature.

In general, analyzes of the changes that have occurred in terms of student performance/learning have been found, or works that focus on the analysis of the anxieties/emotions of teachers during the changes that have occurred since the COVID-19 pandemic; but there are definitely few articles that analyze the emotions linked to the teaching-learning process of reading and writing during this period. It is important to point out that this is not a new element, since it is not a topic that had been widely addressed from investigative coordinates since before the pandemic (Justo, 2004).

In the study by Sánchez Amate et al. (2021) analyzed the effects of COVID-19 in the Learning Process of Primary School Students through a systematic review of the empirical studies carried out on this particular topic. In this way, the research tried to address the repercussions of COVID-19 at the educational level, specifically in the primary education stage, for which it delved into how it has affected the teaching-learning process at these levels in all its complexity. Analysis variables of this process were included, such as the demands of the situation and the challenges of the teachers, the involvement of the families, and the perceptions and repercussions on the performance and socio-educational development of the students. In this case, special emphasis was placed on students with Autism Spectrum Disorder, since they were of particular interest as they are a population especially vulnerable to changes of this magnitude. The research raises as conclusions the existence of a digital gap in certain groups of the child population and a lack of teacher training to deal with situations with these characteristics. These problems hinder the teaching-learning process and contribute to increasing social differences in this population group (Sánchez Amate et al., 2021).

Emotional variables were not analyzed in this study, however the research by Ceballos and Sevilla (2020) was part of the sample, whose objective was to know the effect of social isolation by the COVID-19 on emotional awareness and reading comprehension in students with learning disabilities (ASD and ADHD students). The aforementioned study was developed with a sample of 40 participants to whom interviews were applied and the main results indicate that isolation has a negative emotional impact on students with learning disorders. In the same way, emotional instability is observed in these specific students, which obviously has an impact on the academic performance of the subjects and, therefore, on their reading comprehension abilities.

On the other hand, Cavallo (2021) presents a study in which the objective was to identify the factors that intervene in the teaching and learning process of reading and writing in the context of the COVID-19 pandemic, through the analysis research and theoretical perspectives on this subject. The main conclusion reached is that it is necessary to understand, from a theoretical-interpretative perspective, the factors that currently intervene in the achievement of reading-writing skills and recognize their involvement in the educational process with the main purpose of developing an emerging literacy from the first years of life of boys and girls to enhance these skills.

This is an article that has been of main interest in this analysis as it is the only one found in the analyzed literature review that directly contributes to answering the research questions posed. As limitations in methodological terms, it must be said that this is not an empirical article, but at the same time this is an aspect of value, since its results emerge from a review of the studies carried out on the subject and even an analysis of classical theoretical-methodological references. Thus, the following are defined as elements that have impacted emotions in the teaching-learning process of reading and writing during the pandemic period:

- Socio-economic situation of the families.

Faced with such a far-reaching change situation, it would have been desirable for the students’ families to share knowledge and strategies to enable the development of basic skills of reading-writing, as a way of actively collaborating in the development of these skills and give continuity to the pedagogical work carried out in the classroom at home (Cavallo, 2021) This knowledge and strategies not only concern the purely technical and operational, but also allude to the emotional accompaniment that this process entails (development of sensitivity and the capacity for appreciation, emotional containment, frustration management, etc.) This has not been fulfilled in most cases given the sociocultural, economic, socioeconomic differences, etc. and the wide diversity in these variables that families present today.

- Changes in the modality in which the classes are developed.

On the one hand, Cavallo (2021) states that teachers have had to adapt to the use of new technological tools and this has necessarily meant updating their knowledge to adapt the implementation of methodological strategies and teaching resources to the development of their classes from an inclusive vision, through platforms (ICT) developed for educational purposes. The foregoing places many teachers in a new pedagogical scenario that implies multiple challenges (unawareness of the new tools, technical difficulties inherent to said tools, lack of adequate equipment and the ideal connection speed for these situations, etc.).

In another sense, families have also presented similar problems (lack of technological equipment at home, connection difficulties, etc.).

Therefore, Cavallo (2021) concludes that there is no certainty as to whether the development of significant learning has been successfully achieved in the students, during this period, since the situation described above generates numerous obstacles in the teaching-learning process.

- The type of educational establishment.

Although there are studies (Dronkers and Robert, 2008; Pascual et al., 2018) that suggest that the type of educational establishment (public or private) does not significantly influence the performance of students under normal conditions, in the case of the COVID-19 pandemic could have also influenced the performance and emotional stability of children, especially due to the fact that -in general terms-, private establishments have more technical resources and the students enrolled belong to higher social classes. High with more purchasing power.

- The maturity of children.

The child development process does not take place in the same way in all children, since each one has a certain rhythm and their more or less rapid maturity depends on multiple factors. For the above, Cavallo (2021) draws attention to the fact that the approach must consider individual characteristics, their learning needs and interests, etc. In the case of the COVID-19 pandemic, this was evidently an element that had an impact on the teaching-learning processes of reading and writing.

On the other hand, Thomas (2021) analyzed the performance and self-determined motivation during the closure of schools in 2020 from a quasi-experimental study in a sample of 203 primary school students. The objective of this research was to assess whether there were differences in reading comprehension and self-determined reading motivation of students who attended grades one and two during or before the pandemic. The results were surprising as no differences in reading comprehension and self-determined reading motivation were revealed between the groups, contradicting the initial hypothesis that the pandemic-driven shift to distance learning was a factor that hampered development. of reading comprehension skills and self-determined motivation.

This study also aroused particular interest in view of the objectives of the present investigation since, although the emotions linked to the teaching-learning process of reading-writing were not analyzed, affective variables such as the motivation. Of particular interest is the fact that while the results of this study are encouraging, the research is limited in that it cannot explain what factors contributed to these findings. One possibility in the discussion is that teachers had more time to concentrate on teaching reading; because the school closure meant that students did not participate in non-academic classes (gym, art; Thomas, 2021). On the other hand, the advantage of having smaller classes to work more deeply on reading comprehension is raised (Thomas, 2021). A closer and more personalized attention could influence both academic performance and motivation toward the teaching-learning process (Thomas, 2021).

## Discussion

The objective of this study was to analyze the impact of the socio-psychological changes that have occurred in the educational context since the COVID-19 pandemic on the emotions linked to the teaching-learning process of reading and writing through a systematic review of the studies carried out on the subject; for the identification of recommendations in the development of face-to-face learning in the post-covid era. For which the following research questions were defined:

RQ1: Have the changes that have occurred in educational institutions, derived from the COVID-19 pandemic, influenced the emotions linked to the teaching-learning process of reading and writing?

RQ2: What impact has the COVID-19 pandemic had on emotions linked to the teaching-learning process of reading and writing?

Regarding the first question, it must be said that there are no data that allow us to assert that significant changes have occurred in the emotions linked to learning to read and write specifically. There is, however, research that provides results from which the emotional experience in learning processes can be analyzed during the COVID-19 pandemic at different levels of education. In this case, the research by Zhao and Song (2022) can be cited, where from questions such as what is the emotional experience of students in online learning? Does online learning have the same characteristics as face-to-face learning? Does hybrid learning impact students? How does the emotional response take place in both cases? Therefore, a study was designed to explore the emotional experience of Chinese university students in a context of blended learning (Zhao and Song, 2022). The results showed that students’ emotions in face-to-face classes are more intense than those in online learning, both positive and negative.

When deepening the analysis, very relevant data is found, for example, that in face-to-face teaching the average values of feeling of challenge, comfort, sense of community, satisfaction, enthusiasm and interest are significantly higher than those of online learning. On the other hand, in the case of negative emotions, boredom and disappointment in online learning are more intense than in the case of face-to-face teaching, while stress, embarrassment, tension and frustration in face-to-face learning are significantly more intense. Stronger than online learning (Zhao and Song, 2022). The main result of the study was the identification of 11 factors that influence the emotional state associated with learning. These factors were: degree of difficulty, readiness before class, mastery of knowledge, workload, learning content, teaching paradigm, personal emotion, interaction and collaboration, assessment, peer influence, self-regulation (Zhao and Song, 2022).

The foregoing makes it possible to analyze all the factors involved in learning and that could also participate in the case of reading and writing and make significant differences in face-to-face (F2F) learning with respect to blended learning or purely online teaching.

It would have been desirable to find a study with these characteristics for the case of elementary education where learning to read and write takes place, but it is understandable that since it is such a specific population/topic, despite its high relevance for development socio-emotional of infancies; an investigative interest does not emerge on the part of the hegemonic referents of analysis.

Another study that was also interesting but was not included in this review, since it started from a different population than the one that constituted the specific objective of the study in this case; This is the research developed by Loukomies and Juuti (2021), who took as a reference the period of virtual learning that took place during the confinement to design future strategies for similar occasions, in which adequate support for all students is guaranteed. An instant video blog (IVB) was used to collect reports from primary school students about capturing their emotions in remote learning situations.

Thus, 23 Finnish fifth grade students (11–12 years old) took part in IVB during the remote learning period March 2020 to May 2020. The main and most interesting result of this study is that the expressions of experiences. Negative emotional emotions of the students had a more diverse character than those related to the positive ones (Loukomies and Juuti, 2021). The negative feelings with the greatest presence were boredom and irritation, and the most reported negative aspects related to learning were the difficulty of the tasks or the experience of not having learned anything at all. Toward the end of the research, which coincided with the end of the confinement period, positive emotional expressions related to the face-to-face reunion in the classroom began to emerge (Loukomies and Juuti, 2021).

Another aspect that is not recorded in the studies and that would have been of great relevance is analyzing the influence of the use of a mask on learning to read and write. There are studies that address the influence of mask use on mood and emotion recognition in adolescents and adults (Kulke et al., 2022). In the case of the teaching-learning process of reading and writing, the recognition of emotions plays a very important role not only because of the importance of identifying emotional expressions of frustration, anger, dissatisfaction, etc. in students and provide tools to manage them, but also because the reading-writing process is a process of learning about the world, of reading reality itself, with all the affectivity that this entails. If this process were hindered by the use of a mask, which would be a possible hypothesis, it would be essential to design strategies to counteract this effect on students.

All of the above is consistent with the results shown in the previous section. It is possible to speak of an influence of the changes in educational centers derived from the pandemic on the emotions linked to the teaching-learning processes in general at different levels of education, not only in the case of elementary and primary education. Emotions impacted the teaching-learning process of different subjects and educational levels, and in some cases, this could be seen aggravated by the existing digital gaps in certain population groups and by the gaps in teacher training to deal with these changes. so giddy. It was found that these last two were factors that hindered the teaching-learning process and increased the existing digital gaps (Sánchez Amate et al., 2021).

Specifically, in relation to the second research question (RQ2), it can be said that the COVID-19 pandemic has indeed impacted the emotions linked to the teaching-learning process of reading and writing, influenced by the following factors: social situation -economic of the families, change in the modality in which the classes are developed, type of educational establishment and maturity of the children.

However, the influence on emotions does not seem to have taken place in a negative way in all cases, as expected since there is empirical evidence that there were cases in which no differences were revealed in reading comprehension and self-determined. Reading motivation in learning prior to the pandemic with respect to what happened afterwards. This does not support the hypothesis that the change to distance learning driven by the pandemic was a factor that hindered the development of reading comprehension skills -an essential part of the teaching-learning process of reading-writing and self-determined motivation (Thomas, 2021).

Another important aspect has to do with the management of the situation that took place within the families. Ramkissoon (2020) draws attention to what corresponds to the role of the family -among other contextual elements-, in the real impact that the pandemic had at the individual level. The author explains that the interaction with other members of the household could help create meanings that enhance collective actions that contribute to the development of indicators of psychological well-being (Ramkissoon, 2020). Obviously the meanings shared within family life will condition the responses of all its members, in the case of children this is met with special intensity, in the management of emotions in any scenario, the educational one included.

In summary, although no study has been found that directly addresses the object of this research from an empirical paradigm, therefore it is difficult to answer the research questions raised. However, interesting analysis elements emerge that would be interesting to consider for future designs of blended learning models, online learning focused on reading and writing, and to propose new lines of future research.

## Conclusion

By way of conclusion, it must be said that although it was not possible to analyze the impact of the socio-psychological changes that have occurred in the educational context since the COVID-19 pandemic on the emotions linked to the teaching-learning process of the reading-writing through a systematic review of the studies carried out on the subject; for the identification of recommendations in the development of face-to-face learning in the post-covid era; it has been possible to identify some ideas of interest for future curricular designs in primary school students who are immersed in learning to read and write.

It is important to work on the possible existing digital gaps in the student body in ordinary time, either because of the socioeconomic situation of the families or because of the specific reality of the region. Digital skills today have a primary role in the training of students and in the development of skills for life. In this sense, the implementation of extracurricular programs/projects that contribute to reducing these gaps would be interesting.

Research is necessary to identify/address the experiences and emotional expressions associated with the teaching-learning process of reading and writing. This process constitutes an essential element of the child’s socio-emotional development in the early school stages and it will be very complex to address and teach how to manage these emotions in the classroom if there is no clarity regarding the specific mechanisms related to this particular learning. This could be channeled through the school curriculum itself through various creative strategies from the methodological modality of action research.

The possibilities offered by online and hybrid teaching-learning designs, in terms of student reach, are endless and certainly implementing curricular designs focused on reading and writing is highly complex given the nature of said learning process and the characteristics of the evolutionary stage in which it occurs. However, a fundamental element that will be essential to consider is the direct work between teachers and students. If this link is developed properly, it can contribute significantly to the reduction and healthy management of intragroup conflicts in class, to the increase in academic performance and to the adequate management of emotions related to the teaching-learning process.

Regarding the limitations of this research, from the methodological point of view, the limitations of the systematic review can be mentioned, such as publication bias, since there may be a significant number of studies that were not published due to did not reach statistical significance or even because there were no resources for its publication in the investigated databases. Likewise, another limitation in this case and that is inherent to systematic reviews is their retrospective nature, so that the readings made of the results of the studies analyzed could be biased by interpretations outside the context in which the studies were carried out. Themselves.

Regarding the theoretical limitations, the specificity and particularity of the topic that was intended to be addressed should be mentioned, which significantly limited the search and undoubtedly influenced the identification of relevant studies that contributed to answering the research questions posed.

As lines of future research, the design of studies that allow addressing the objectives of this study from an empirical perspective could be considered. Another line that could be derived from the present study with the aim of compensating for the limitations found in the research process would be to change the scope of the objective and expand it toward, for example, the analysis of emotions in the period of elementary and/or primary education during the COVID-19 pandemic in its relation to the academic performance of students.

## Author contributions

MM-G contributed in review analysis and wrote the paper. AB and CL-G wrote the paper. SD collected the data. SB and JE contributed in review analysis. All authors contributed to the article and approved the submitted version.

## Funding

This study was funded by Comunidad de Madrid, Atracción de Talento modalidad 1 (grant number 2018-T1/SOC-10409), and MM-G was supported by the Ministerio de Universidades and the European Union “NextGeneration EU/PRTR” through 2021-2023 Margarita Salas.

## Conflict of interest

The authors declare that the research was conducted in the absence of any commercial or financial relationships that could be construed as a potential conflict of interest.

## Publisher’s note

All claims expressed in this article are solely those of the authors and do not necessarily represent those of their affiliated organizations, or those of the publisher, the editors and the reviewers. Any product that may be evaluated in this article, or claim that may be made by its manufacturer, is not guaranteed or endorsed by the publisher.
